# The internal structure of foster-parent completed SDQ for school-aged children

**DOI:** 10.1371/journal.pone.0176625

**Published:** 2017-06-30

**Authors:** Stine Lehmann, Tormod Bøe, Kyrre Breivik

**Affiliations:** Uni Research Health, Regional Centre for Child and Youth Mental Health and Child Welfare, West, Bergen, Norway; Swinburne University of Technology, AUSTRALIA

## Abstract

Mental health problems are common in foster-children, and tools to measure the mental health of these children are needed. One candidate instrument is the Strengths and Difficulties Questionnaire (SDQ), a measure of child psychological adjustment that is increasingly being employed by Child Protection services. The aim of the current study was to examine the structural validity of the foster parent completed SDQ in a sample of 237 school aged foster children. Confirmatory factor analysis demonstrated an excellent fit of the foster parent completed SDQ data to a five-factor model (CFI = 0.96, RMSEA = 0.05, 90% CI [0.04, 0.06]), thus confirming the structural validity of the five-factor model for the parent-version of the SDQ in Norwegian foster children. Measurement invariance analyses indicated that boys had lower thresholds for fighting with or bullying other children than girls. Girls were on their side more likely to be rated as less popular than boys with a similar level of peer problems.

## Introduction

The high prevalence and comorbidity of mental disorders in foster children [1–3] highlight the need to examine and monitor the mental health of children living in foster homes. Child protection services often have limited competence and resources for conducting in-depth assessments of mental health, making brief screening instruments an attractive option.

The Strengths and Difficulties Questionnaire (SDQ) [4] is a commonly used screening-instrument of psychological adjustment in children and adolescents. The present study examine the internal structure of the five-factor model proposed by Goodman [5].

Since its development, the SDQ has been employed in epidemiological and community surveys, and it is commonly used in clinical practice [6–11]. The validity and clinical utility of the SDQ has been demonstrated in several previous investigations [6, 10, 11]. The SDQ has good concurrent and discriminant validity, and correlates strongly with related scales such as the Youth Self-Report and the Revised Children’s Manifest Anxiety Scale [12–16], it discriminates well between children with and without mental problems [4, 5], it may be used to effectively screen for disorders in community samples [17], and is adequately sensitive to serve as a clinical outcome measure [18]. SDQ has also proven good predictive ability in British looked-after children (n = 1391, of whom 38.6% had a mental disorder;[19]), and a general child protection sample (n = 292, 29% of these had contact with mental health care; [20]).

Prior studies of the internal structure of the parent version of the SDQ using confirmatory factor analysis [21–26] have tended to support the original five factor model proposed by Goodman [5]. The still limited amount of research indicates that the Parent SDQ is measurement invariant across gender [22, 26, 27].

Studies in the UK and Canada have demonstrated that the SDQ can be useful for screening, in samples of looked after children [28, 29]. The screening properties of the SDQ has also been demonstrated in a previous study with the same sample as in the current study [30].

The use of the SDQ in child protection contexts is increasing both for research and clinical purposes [31–36]. In the UK, the government has put in place mandatory administration of the SDQ by all primary carers of looked after children [37].

Investigations of the validity of the SDQ, as with the five-factor structure suggested by previous studies of community-samples, are lacking for foster children. This is a significant limitation of the knowledgebase on the suitability of SDQ as a screening instrument for this higher risk population. A previous study has demonstrated a comorbidity-rate for foster children that are significantly higher than that found in both community samples and youths referred to CAMHS [38]. The substantial cross-diagnostic symptom-overlap in this group may decrease the fit of a model hypothesizing five discrete factors of the SDQ when used for this high risk group of children. Palmieri and Smith [22] found support for the five-factor structure of the SDQ in a sample of 773 custodial grandparents. The most common placement form is however placement in foster families consisting of non-biological primary carers. The present study is the first study to investigate if this factor-structure of the SDQ is still valid when regular foster parents are informants.

The aims of the current study were 1) to examine the internal structure of the SDQ in a sample of Norwegian foster parents, and 2) to evaluate whether the SDQ was measurement invariant across gender.

## Materials and methods

### Participants

Eligible participants were foster children between the age of 6 and 12 years who had lived for at least 5 months in foster families in the 63 municipalities encompassed by the Regional Office for Children, Youth and Family Affairs -South (Bufetat). The regional register at Bufetat listed 391 eligible children, but an additional 28 were identified and added after the office heads of each municipal child protection services revised the lists. Twenty children who had been returned to their biological families, or who had been adopted were excluded from the list. Further, three children were deemed ineligible because of serious neurological disabilities, resulting in a total of 396 children being eligible for inclusion in the study. The current study included 237 children (47.7% girls, mean age = 8.95, *SD* 2.04) with SDQ completed by foster parents. Analyses revealed that foster fathers and–mothers responded similarly [30], and they were therefore combined into a single group of informants, hereafter referred to as “caregivers”, prioritising information from the foster mothers (n = 213) when available, resulting in 20 father completed SDQs. Of the 237 children with caregiver-completed SDQs, 168 children had a custom made Child Protection Questionnaire completed by the caseworker. Independent samples t-tests showed no difference between the children with and without CPQ information (n = 69), regarding sex, age or scores on the SDQ Total Difficulties- and subscales. For the subsample with CPQ completed, mean age at first placement was 3.43 (*SD* 3.07); mean sum of previous placements was 1.23 (*SD* 1.11); the children had stayed from 5 months to 12 years with their current foster family (mean 4.51, *SD* 3.18).

### Measures

The SDQ is a 25-item mental health questionnaire for 3- to 16-year-olds [39]. Versions of SDQ exist for parents and teachers of children aged 4–16, and as a self-report version for children aged 11–16. It consists of a Prosocial subscale, a Peer Problems subscale and three symptom subscales; Emotional Symptoms, Conduct Problems and Hyperactivity-Inattention. Each subscale consists of five items that are rated on a three-point-scale (0-1-2), adding up to a subscale-score ranging from 0–10. The scores on the subscales (excluding prosocial behaviours) may further be added into a Total Difficulties score, with a range from 0–40. The SDQ also includes an Impact scale, measuring distress to the child and the extent of which symptoms and problems interfere with the child’s daily life [40]. Impact scales are included in both parent (five items, score range 0–10) and teacher (three items, score range 0–6) versions of the instrument.

A Child Protection Questionnaire (CPQ) developed for this study. This questionnaire assessed the placement history of the child, i.e. time in current foster home, age at first placement and number of previous placements.

### Procedure

Data collection started on September1^st^ 2011, and lasted until the end of February 2012. The caseworker in the municipal child protection service received a postal letter with detailed information about the study and was asked to complete and return the CPQ to the principal investigator. Foster parents received a postal letter with detailed information about the study, and instructions on how to complete the SDQ online.

### Ethics

The study was approved by the Regional Committee for Medical and Health Research Ethics in Western Norway. In accordance with Norwegian ethics requirements, children 12 years and older gave assent. According to Norwegian legislation, foster parents do not have the mandate to consent on behalf of their foster children. The study was therefore reviewed by the Ministry of Children, Equality and Social Inclusion, who provided caseworkers and foster parents with exemption from confidentiality for the current study.

### Statistical methods

In estimating reliability of the five subscales in the SDQ, Stone and Otten [26] recommends the use of omega alpha coefficient (ω_h)_, based on a factor model with one factor, where _h_ relates to the hierarchical or general factor, as described in Mc Donald [41]. We employed the procedure as described in Stone, and calculated the reliability of each factor in the five-factor model.

Confirmatory factor analysis (CFA) based on data from foster parents was performed using Mplus 7.1 [42]. The CFA models were estimated using a robust weighted mean and variances adjusted least squares estimator (WLSMV) with DELTA parameterization, to account for the multivariate non-normality (Mardia’s coefficient (61) of multivariate kurtosis, 705.392, p <.001; multivariate skewness, 101.626, p <.001) and the categorical data (ordinal data with three options). Using polychoric correlations for estimation, the WLSMV appear relatively robust to violations of normality [43, 44]. One influential outlier (Cook’s d = 1.65) were identified, and removed prior to the CFA analyses, reducing sample size to N = 236.

It was hypothesized that the data would fit a five-factor structure, corresponding to the five subscales. Indicators for the 5 factors are illustrated in Table 1. The fit of the CFA model was evaluated according to the following standard fit indices [45]: The Comparative fit index (CFI) is an incremental model fit index, with value ranging from 0 to 1. The Root Mean Square Residual (RMSEA) is an absolute index of fit and determines how well the hypothesized model fit the data. It is sensitive to the number of parameters in the model. The recommended cut-offs for good fit are CFI ≥ .95, and RMSEA < .06 when using the WLSMV estimator [46].

**Table 1:** Factor loadings (standardized) for the modified^a^ five latent factors in the P-Strengths and Difficulties Questionnaire (N = 236).

		F1	F2	F3	F4	F5
	M (SD)	Est (SE)	Est (SE)	Est (SE)	Est (SE)	Est (SE)
SDQ Emotional problems						
Somatic	0.44 (0.69)	.49 (.08)				
Worries	0.64 (0.73)	.78 (.05)				
Unhappy	0.56 (0.69)	.77 (.05)				
Clingy	1.00 (0.83)	.52 (.07)				
Afraid	0.80 (0.80)	.63 (.06)				
SDQ Conduct problems						
Tantrum	0.78 (0.78)		.81 (.04)			
**Obeys**	0.80 (0.66)		.71 (.05)			
Fights	0.28 (0.55)		.74 (.05)			
Lies	0.82 (0.76)		.68 (.05)			
Steals	0.26 (0.56)		.66 (.06)			
SDQ Hyperactivity-/inattention						
Restless	0.90 (0.78)			.70 (.05)		
Fidgety	0.98 (0.80)			.84 (.04)		
Distracts	1.43 (0.74)			.72 (.06)		
**Reflect**	1.42 (0.64)			.74 (.05)		
**Attend**	1.32 (0.73)			.65 (.06)		
SDQ Peer problems						
Loner	0.54 (0.69)				.67 (.06)	
**Friend**	0.60 (0.76)				.51 (.07)	
**Popular**	0.59 (0.62)				.74 (.06)	
Bullied	0.57 (0.56)				.72 (.06)	
Oldbest	0.69 (0.78)				.74 (.05)	
SDQ Prosocial behaviors						
Considerate	1.19 (0.66)					.95 (.04)
Shares	1.37 (0.67)					.76 (.06)
Caring	1.41 (0.67)					.78 (.04)
Kind	1.74 (0.47)					.68 (.06)
Helpout	1.11 (0.71)					.42 (.07)

*Note*. Items in bold are reverse coded.

^a^ The modified model included correlations between (the error terms of) Fidgety and Restless (r = .69), Attend and Distracts (r = .63), Afraid and Clingy (r = .57).

To test whether the model was representative for both boys and girls we first tested the model separately for these two groups. In order to test for measurement invariance in factor loadings and thresholds across gender we used the automated Measurement Invariance routine for testing Configural, Metric (equal factor loadings) and Scalar invariance (equal factor loadings and thresholds) between groups available in Mplus [47]. These traditional invariance analyses were followed up by testing whether potential local dependencies (correlated residuals), and associations between factors and means were invariant across gender. An adjusted chi-square test (DIFFTEST) was used to test whether the nested models differed significantly from each other as it is not proper to use a traditional Δχ test when using the WLMSV estimator [48]. The model was assumed non-invariant if the adjusted chi-square was significant and the change in CFI was < = −0.002 [49].

## Results

Mean Total Difficulties score was 15.21 (*SD* 7.90); 3.46 (*SD* 2.57) on Emotional subscale; 2.94 (*SD* 2.32) on Conduct subscale; 6.05 (*SD* 2.82) on Hyperactivity subscale; 2.76 (*SD* 2.31) on Peer problem subscale; 6.82 (*SD* 2.30) on Prosocial subscale; and 2.67 (*SD* 2.72) on the Impact scale. There were no missing values in the parent completed SDQs.

### Internal reliability

The ω coefficients derived from the results of the CFA showed acceptable to high reliability for Emotional Symptoms (.78), Conduct Problems (.84), Hyperactivity-Inattention (.85), Peer Problems (.81), and Prosocial Behaviour (.85).

### Confirmatory factor analyses

The original five-factor model showed an acceptable, but not excellent, fit to our data (*X*^2^ = 500.15, *df* = 265, *p* < 0.001, CFI = 0.94, TLI = 0.93, RMSEA 0.06, 90% Confidence Interval [CI] [0.05, 0.07]). Modification indices suggested that model fit could be improved by accounting for some correlated error terms in items in the hyperactivity-/inattention factor (i.e. Fidgety with Restless, Attends with Distract) and in the emotional problems factor (Afraid with Clingy). The revised model showed an excellent fit to the data (*X*^2^ = 401.81, *df* = 262, *p* < 0.001, CFI = 0.97, TLI = 0.96, RMSEA = 0.05, 90% CI [0.04, 0.06]). Table 1 shows the factor loadings for the modified five latent factors in the SDQ, parent version.

Table 2 shows the trait correlations between the five factors in the model. These were generally high ranging from (-).46 between Prosocial and Emotional problems to .84 between Hyperactive and Conduct. Polychoric correlations between items in the SDQ are included in S1 Table. Even if the aim of the paper was to confirm the structural validity of the 5 factor solution in this sample of foster children, we also tested the fit of a 4 factor solution where the Hyperactivity and Conduct factors where treated as one factor due to their high correlation. The 4 four factor solution had however a significantly poorer fit than the five factor solution (Δ*X*^2^ = 13.158, Δ*df* = 4, *p* < 0.05).

**Table 2:** Correlations between latent factors of the Strengths and Difficulties Questionnaire (N = 236).

Factor	F1	F2	F3	F4	F5
F1 SDQ Emotional Symptoms	1.000				
F2 SDQ Conduct Problems	.72	1.000			
F3 SDQ Hyperactivity-Inattention	.70	.84	1.000		
F4 SDQ Peer Problems	.67	.79	.71	1.000	
F5 SDQ Prosocial	-.46	-.69	-.55	-.47	1.000

### Measurement invariance analyses

The revised five-factor model was tested separately for boys and girls and had an adequate and very similar fit in both groups supporting that the five factor structure has an adequate fit to data for both sexes (boys, n = 124: *X*^2^ = 327.64, *df* = 262, p < 0.001, TLI = 0.96, CFI = 0.97, RMSEA = 0.05, CI [0.03, 0.06]; girls, n = 112: *X*^2^ = 321.94, p < 0.001, df = 262, TLI = 0.97, CFI = 0.97, RMSEA = 0.04, CI [0.02–0.06]). The remaining measurement invariance analyses are shown in Table 3.

**Table 3:** Testing Measurement Invariance of the Strengths and Difficulties Questionnaire factors across gender.

	Model development				Model comparison				
	*df*	*Chi-square*	*CFI*	*RMSEA*	Comparison	*Δdf*	*ΔChi square*^+^	*p-*value	*ΔCFI*
M1: Configural invariance	522	648.566***	0.966	0.045 (0.033–0.056)	-	-	-	-	-
M2: Metric invariance	542	663.121***	0.968	0.043 (0.031–0.054)	M2 versus M1	20	17.101	0.65	0.002
M3: Scalar invariance	562	690.120***	0.966	0.044 (0.031–0.055)	M3 versus M1	40	49.499	0.14	0.000
					M3 versus M2	20	32.230	0.04	-0.002
M4: Partial Scalar invariance^†^	560	681.638***	0.967	0.043 (0.030–0.054)	M4 versus M2	18	23.781	0.16	-0.001
M5: M4 + equal local dependencies	564	686.899***	0.967	0.043 (0.030–0.054)	M5 versus M4	4	6.487	0.17	0.000
M6: M5+ equal factors correlations^‡^	574	697.041***	0.967	0.043 (0.030–0.053)	M6 versus M5	10	14.345	0.16	0.000
M7: M6 + equal means^‡^	579	691.309***	0.970	0.040 (0.027–0.052)	M7 versus M6	5	3.062	0.69	0.003

Note.

^†^ The thresholds of the Popular and Fights items were allowed to be freely estimated for boys and girl in this and the following models;

^‡^ The residual covariance matrix was not positive definite in the girls group;

^+^ Tested using DIFFTEST in Mplus;

***p < .001

The measurement invariance analyses supported scalar invariance, as the (adjusted) Δ *X*^2^ between comparisons between the three first models was non-significant. None of the other model comparisons resulted in a statistically significant Δχ, indicating that the size of the local dependencies, covariance between factors and factor means do not differ for boys and girls in this sample. A potential problem with the approach we have used for detecting measurement invariance, is that is based on model comparisons of a global fit measure (delta chi square) which in some situations might not be sensitive enough to detect local misspecifications. To locate such potential local misfits we examined whether any of the modification indexes of the scalar model (M3) were significant and had a standardized EPC (expected factor loading or threshold change) larger than 0.1. This search revealed that somatic symptoms might be more discriminative (stronger factor loading) and worries less discriminative for boys than girls with regard to Emotional symptoms factor. Steals might be somewhat more discriminative and fights less discriminative for girls compared to boys with regard to conduct problems. Finally, being popular might be more discriminative (and thus provide more information) for girls than boys with regard to peer problems. These local misfits are included as potential problems which have to be replicated by other studies before they are given any weight, as there is a high risk of capitalizing on chance given the low sample size in the present study, their explorative nature, and the very large number of parameters that have been tested.

## Discussion

The findings in this study support the 5-factor structure of the SDQ when completed by foster parents of school-aged children. The fit of the original five factor model was acceptable, but could be improved by accounting for certain local dependencies (correlated error terms). Two local dependencies within the hyperactivity/inattention factor where discovered. These local dependencies probably represent minor hyperactivity (“Fidgety” and “Restless”) and Inattention (“Attends” and “Distracts”) factors within the broader hyperactivity/inattention factor. Similar local dependencies (especially between “Fidgety” and “Restless”) within the hyperactivity/inattention factor have been found in several other studies [23, 25, 50–52]. These findings are in line with a growing number of studies that have found support for a bifactor model representation of ADHD symptoms [53]. This model hypothesize that ADHD symptoms are best explained by both a general ADHD factor as well as domain specific factors related to hyperactivity/impulsivity and inattention. In the present study, a local dependency between the items “Afraid” and “Clingy” within the emotional problems factor was also discovered. This particular local dependency has been reported in at least two previous studies [23, 54] and we hypothesize it represents a more narrow anxiety factor within the broader emotional problems factor. Future CFA studies of the SDQ should in our opinion take these local dependencies into account to give a more complete representation of broad and narrow factors that have an impact on item responses.

The correlations between latent factors were generally rather large, and often larger than what have typically between reported in previous studies [55, 56]. In fact the correlation between the Conduct and the Hyperactivity factors nearly equals the often used cut off criterion (r = .85) for problematic discriminative validity [57]. The unusually large correlations between the factors in this study probably reflects the often high comorbidity of mental disorders in foster children [38]. And even if the correlation between Conduct and Hyperactivity is very large we believe that they should be still be treated as two separate factors in future research as they have shown to show discriminant validity with respect to clinical disorder [54].

All the items had moderate to high factor loadings on their respective factors. Using the results from the modified CFA, reliability analyses indicated that all SDQ scales had an acceptable to very good reliability, as estimated by omega.

Overall, the 5 factor model was representative for both boys and girls when testing the model separately for the two groups.

To our knowledge, this is the first study examining the factor structure of the SDQ for foster parents. Therefore, our findings add substantially to the knowledge of the feasibility of SDQ as a screening measure in child protection services. Our findings strengthen the rationale for use of the SDQ in child protection services, where caregiver information may come from foster parents rather than biological parents. This is important, as information about child mental health may be difficult to obtain from biological parents in a child protection context.

### Study limitations

There are several limitations to the study that needs to be addressed. We did not obtain information on whether the foster parents in this study were kin to the child or not. As 26% of children placed in foster families during 2013 were placed in kin or close network [58], we must assume that a substantial proportion of the foster parents in this sample know the child from close family and social relation also before placement. This group may closely resemble biological parents in their knowledge of the children’s mental health and development.

The limited sample put restrictions on the forms of analyses possible. The power of the models in this study is rather low and the parameter to case ratio is as low as 1.34 (236/176) for the configural model. Therefore, results should be interpreted with caution.

## Conclusions

The present findings support the use of the SDQ congruent with the suggested five-factor structure, also in child protection services. Together with previous findings of good predictive properties of the SDQ for school-aged foster children [30], these results indicate that SDQ may be used to screen foster children for mental health problems. Still, caution should be taken against using the SDQ as the sole instrument to screen for mental health and relational functioning, as the construct of Reactive Attachment disorder and Disinhibited Social Engagement Disorder seem salient in the description of these children’s relational malfunctioning [59]. Further research is needed to gain more knowledge about how to best combine scales from screening instruments to adequately capture dimensions of psychopathology especially relevant for this high-risk group of children.

## Supporting information

S1 Table Polychoric correlation matrix between items in the SDQ. (DOCX)

## References

[1] Ford T, Vostanis P, Meltzer H, Goodman R. Psychiatric disorder among British children looked after by local authorities: Comparison with children living in private households. British Journal of Psychiatry. 2007;190 (APR.):319–25. 10.1192/bjp.bp.106.025023. 17401038

[2] McMillen JC, Zima BT, Scott LD Jr, Auslander WF, Munson MR, Ollie MT, et al Prevalence of psychiatric disorders among older youths in the foster care system. J Am Acad Child Adolesc Psychiatr. 2005;44 (1):88–95. 10.1097/01.chi.0000145806.24274.d2. 15608548

[3] Lehmann S, Havik O, Havik T, Heiervang E. Mental disorders in foster children: a study of prevalence, comorbidity and risk factors. Child and Adolescent Psychiatry and Mental Health. 2013;7(1):39 10.1186/1753-2000-7-39 24256809 PMC3922948

[4] Goodman R. The Strengths and Difficulties Questionnaire: A research note. Journal of Child Psychology and Psychiatry. 1997;38(5):581–6. 10.1111/j.1469-7610.1997.tb01545.x 9255702

[5] Goodman R. Psychometric Properties of the Strengths and Difficulties Questionnaire. Journal of the American Academy of Child & Adolescent Psychiatry. 2001;40(11):1337–45. 11699809 10.1097/00004583-200111000-00015

[6] Obel C, Heiervang E, Rodriguez A, Heyerdahl S, Smedje H, Sourander A, et al The Strengths and Difficulties Questionnaire in the Nordic countries. Eur Child Adolesc Psychiatry. 2004;13 Suppl 2:II32–9. 10.1007/s00787-004-2006-2 . 15243784

[7] National Center for Health Statistics. National Health Interview Survey (NHIS): Hyattsville, MD: U.S. Centers for Disease Control and Prevention; 2003.

[8] Goodman R, Renfrew D, Mullick M. Predicting type of psychiatric disorder from Strengths and Difficulties Questionnaire (SDQ) scores in child mental health clinics in London and Dhaka. Eur Child Adolesc Psychiatry. 2000;9(2):129–34. 10926063 10.1007/s007870050008

[9] Mathai J, Anderson P, Bourne A. Comparing psychiatric diagnoses generated by the Strengths and Difficulties Questionnaire with diagnoses made by clinicians. Aust N Z J Psychiatry. 2004;38(8):639–43. 10.1080/j.1440-1614.2004.01428.x . 15298587

[10] Woerner W, Fleitlich-Bilyk B, Martinussen R, Fletcher J, Cucchiaro G, Dalgalarrondo P, et al The Strengths and Difficulties Questionnaire overseas: Evaluations and applications of the SDQ beyond Europe. European Child & Adolescent Psychiatry. 2004;13(2):ii47–ii54. 10.1007/s00787-004-2008-0 15243786

[11] Marzocchi G, Capron C, Di Pietro M, Duran Tauleria E, Duyme M, Frigerio A, et al The use of the Strengths and Difficulties Questionnaire (SDQ) in Southern European countries. European Child & Adolescent Psychiatry. 2004;13(2):ii40–ii6. 10.1007/s00787-004-2007-1 15243785

[12] Klasen H, Woerner W, Wolke D, Meyer R, Overmeyer S, Kaschnitz W, et al Comparing the German Versions of the Strengths and Difficulties Questionnaire (SDQ-Deu) and the Child Behavior Checklist. European Child & Adolescent Psychiatry. 2000;9(4):271-. Language: English. Entry Date: 20070101. Revision Date: 20091218. Publication Type: journal article. 11202102 10.1007/s007870070030

[13] Koskelainen M, Sourander A, Kaljonen A. The Strengths and Difficulties Questionnaire among Finnish school-aged children and adolescents. Eur Child Adolesc Psychiatry. 2000;9(4):277–84. . 11202103 10.1007/s007870070031

[14] Muris P, Meesters C, van den Berg F. The Strengths and Difficulties Questionnaire (SDQ)—further evidence for its reliability and validity in a community sample of Dutch children and adolescents. Eur Child Adolesc Psychiatry. 2003;12(1):1–8. 10.1007/s00787-003-0298-2 12601558

[15] van Widenfelt BM, Goedhart AW, Treffers PDA, Goodman R. Dutch version of the Strengths and Difficulties Questionnaire (SDQ). European Child & Adolescent Psychiatry. 2003;12(6):281–9. 10.1007/s00787-003-0341-3 14689260

[16] Yao S, Zhang C, Zhu X, Jing X, McWhinnie CM, Abela JR. Measuring adolescent psychopathology: psychometric properties of the self-report strengths and difficulties questionnaire in a sample of Chinese adolescents. The Journal of adolescent health: official publication of the Society for Adolescent Medicine. 2009;45(1):55–62. 10.1016/j.jadohealth.2008.11.006 . 19541250

[17] Goodman R, Ford T, Simmons H, Gatward R, Meltzer H. Using the Strengths and Difficulties Questionnaire (SDQ) to screen for child psychiatric disorders in a community sample. Int Rev Psych. 2003;15(1–2):166–72. 10.1080/0954026021000046128 12745328

[18] Mathai J, Anderson P, Bourne A. Use of the Strengths and Difficulties Questionnaire as an Outcome Measure in a Child and Adolescent Mental Health Service. Australasian Psychiatry. 2003;11(3):334–7. 10.1046/j.1440-1665.2003.00544.x

[19] Stringaris A, Goodman R. The Value of measuring impact alongside symptoms in children and adolescents: A longitudinal assessment in a community sample. J Abnorm Child Psychol. 2013:1–12. 23677767 10.1007/s10802-013-9744-x PMC3755220

[20] Janssens A, Deboutte D. Screening for psychopathology in child welfare: the Strengths and Difficulties Questionnaire (SDQ) compared with the Achenbach System of Empirically Based Assessment (ASEBA). European Child & Adolescent Psychiatry. 2009;18(11):691–700. 10.1007/s00787-009-0030-y 19462154

[21] Becker A, Woerner W, Hasselhorn M, Banaschewski T, Rothenberger A. Validation of the parent and teacher SDQ in a clinical sample. European Child & Adolescent Psychiatry. 2004;13(2):ii11–ii6. 10.1007/s00787-004-2003-5 15243781

[22] Palmieri PA, Smith GC. Examining the structural validity of the Strengths and Difficulties Questionnaire (SDQ) in a U.S. sample of custodial grandmothers. Psychological Assessment. 2007;19(2):189–98. 10.1037/1040-3590.19.2.189 17563200 PMC1997309

[23] Sanne B, Torsheim T, Heiervang E, Stormark KM. The Strengths and Difficulties Questionnaire in the Bergen Child Study: A conceptually and methodically motivated structural analysis. Psychological Assessment. 2009;21(3):352–64. 10.1037/a0016317 19719347

[24] Van Leeuwen K, Meerschaert T, Bosmans G, De Medts L, Braet C. The strengths and difficulties questionnaire in a community sample of young children in flanders. European Journal of Psychological Assessment. 2006;22(3):189–97. 10.1027/1015-5759.22.3.189

[25] Van Roy B, Veenstra M, Clench-Aas J. Construct validity of the five-factor Strengths and Difficulties Questionnaire (SDQ) in pre-, early, and late adolescence. Journal of Child Psychology and Psychiatry. 2008;49(12):1304–12. 10.1111/j.1469-7610.2008.01942.x 19120709

[26] Stone LL, Otten R, Ringlever L, Hiemstra M, Engels RCME, Vermulst AA, et al The parent version of the Strengths and Difficulties Questionnaire: Omega as an alternative to alpha and a test for measurement invariance. European Journal of Psychological Assessment. 2013;29(1):44–50. 10.1027/1015-5759/a000119

[27] Hill CR, Hughes JN. An examination of the convergent and discriminant validity of the Strengths and Difficulties Questionnaire. School Psychology Quarterly. 2007;22(3):380 10.1037/1045-3830.22.3.380 18843384 PMC2562744

[28] Goodman R, Ford T, Corbin T, Meltzer H. Using the Strengths and Difficulties Questionnaire (SDQ) multi-informant algorithm to screen looked-after children for psychiatric disorders. European Child & Adolescent Psychiatry. 2004;13(Suppl2):II25–II31. 10.1007/s00787-004-2005-3. Peer Reviewed Journal: 2004-17755-005. 15243783

[29] Marquis RA, Flynn RJ. The SDQ as a mental health measurement tool in a Canadian sample of looked-after young people†. Vulnerable Children and Youth Studies. 2009;4(2):114–21.

[30] Lehmann S, Heiervang ER, Havik T, Havik OE. Screening Foster Children for Mental Disorders: Properties of the Strengths and Difficulties Questionnaire. PLoS ONE. 2014;9(7):e102134 10.1371/journal.pone.0102134 25006669 PMC4090209

[31] Richards L, Wood N, Ruiz-Calzada L. The mental health needs of looked after children in a local authority permanent placement team and the value of the Goodman SDQ. Adoption & Fostering. 2006;30(2):43–52.

[32] Callaghan J, Young B, Pace F, Vostanis P. Evaluation of a new mental health service for looked after children. Clinical Child Psychology and Psychiatry. 2004;9(1):130–48.

[33] Iversen AC, Jakobsen R., Havik T., Hysing M., Stormark K.M. Mental healthproblems among Child Wellfare clients living at home. Child Care in Practice 2007;13(4):387–99.

[34] Minnis H, Everett K, Pelosi AJ, Dunn J, Knapp M. Children in foster care: Mental health, service use and costs. European Child & Adolescent Psychiatry. 2006;15(2):63–70. 10.1007/s00787-006-0452-8. Peer Reviewed Journal: 2006-04942-001. 16523249

[35] Teggart T, Menary J. An investigation of the mental health needs of children looked after by craigavon and banbridge health and social services trust. Child Care in Practice. 2005;11(1):39–49. 10.1080/1357527042000332781

[36] Aguilar-Vafaie ME, Roshani M, Hassanabadi H, Masoudian Z, Afruz GA. Risk and protective factors for residential foster care adolescents. Children and Youth Services Review. 2011;33(1):1–15.

[37] Goodman A, Goodman R. Strengths and Difficulties Questionnaire scores and mental health in looked after children. The British Journal of Psychiatry. 2012;200(5):426–7. 2012-21520-014. 10.1192/bjp.bp.111.104380 22550331

[38] Lehmann S, Havik OE, Havik T, Heiervang E. Mental disorders in foster children: a study of prevalence, comorbidity and risk factors. Child and Adolescent Psychiatry and Mental Health. 2013;7(39). http://dx.doi.org10.1186/1753-2000-7-39. 10.1186/1753-2000-7-39 PMC3922948 24256809

[39] Goodman R, Meltzer H, Bailey V. The Strengths and Difficulties Questionnaire: A pilot study on the validity of the self-report version. European Child & Adolescent Psychiatry. 1998;7(3):125–30. 9826298 10.1007/s007870050057

[40] Goodman R. The extended version of the Strengths and Difficulties Questionnaire as a guide to child psychiatric caseness and consequent burden. Journal of Child Psychology and Psychiatry. 1999;40(5):791–9. 10.1111/1469-7610.00494 10433412

[41] McDonald RP. Generalizability in Factorable Domains: "Domain Validity and Generalizability"1. Educational and Psychological Measurement. 1978;38(1):75–9. 10.1177/001316447803800111

[42] Muthén LK, Muthén B. Mplus. The comprehensive modelling program for applied researchers: user’s guide. 5 Los Angeles: Muthén & Muthén; 2012.

[43] Dumenci L, Achenbach TM. Effects of estimation methods on making trait-level inferences from ordered categorical items for assessing psychopathology. Psychological Assessment. 2008;20(1):55–62. 10.1037/1040-3590.20.1.55 18315399

[44] Flora DB, Curran PJ. An empirical evaluation of alternative methods of estimation for confirmatory factor analysis with ordinal data. Psychological Methods. 2004;9(4):466 10.1037/1082-989X.9.4.466 15598100 PMC3153362

[45] Jackson DL, Gillaspy JA Jr, Purc-Stephenson R. Reporting practices in confirmatory factor analysis: An overview and some recommendations. Psychological Methods. 2009;14(1):6–23. 10.1037/a0014694 19271845

[46] Yu CY, Muthen BO. Evaluation of model fit indices for latent variable models with categorical and continuous outcomes. Los Angeles: University of California at Los Angeles, Graduate School of Education and Information Studies; 2002.

[47] Muthén L, Muthén B. Version 7.1 Mplus Language Addendum. https://www.statmodel.com/download/Version7.1x-Language.pdf; 2013.

[48] Muthén LM. Mplus user's guide (7th ed.). Los Angeles, CA 1998-2012.

[49] Meade AW, Johnson EC, Braddy PW. Power and sensitivity of alternative fit indices in tests of measurement invariance. J Appl Psychol. 2008;93(3):568 10.1037/0021-9010.93.3.568 18457487

[50] Ronning JA, Handegaard BH, Sourander A, Morch W. The Strengths and Difficulties Self-Report Questionnaire as a screening instrument in Norwegian community samples. European Child & Adolescent Psychiatry. 2004;13(2):73–82. Language: English. Entry Date: 20070101. Revision Date: 20091218. Publication Type: journal article. 15103532 10.1007/s00787-004-0356-4

[51] Percy A, McCrystal P, Higgins K. Confirmatory factor analysis of the adolescent self-report Strengths and Difficulties Questionnaire. European Journal of Psychological Assessment. 2008;24(1):43–8.

[52] van de Looij-Jansen PM, Goedhart AW, de Wilde EJ, Treffers PD. Confirmatory factor analysis and factorial invariance analysis of the adolescent self-report Strengths and Difficulties Questionnaire: How important are method effects and minor factors? British Journal of Clinical Psychology. 2011;50(2):127–44. 10.1348/014466510X498174 21545447

[53] Toplak ME, Sorge GB, Flora DB, Chen W, Banaschewski T, Buitelaar J, et al The hierarchical factor model of ADHD: invariant across age and national groupings? Journal of Child Psychology and Psychiatry. 2012;53(3):292–303. 10.1111/j.1469-7610.2011.02500.x 22084976 PMC3272099

[54] Goodman A, Lamping DL, Ploubidis GB. When to use broader internalising and externalising subscales instead of the hypothesised five subscales on the Strengths and Difficulties Questionnaire (SDQ): data from British parents, teachers and children. J Abnorm Child Psychol. 2010;38(8):1179–91. 10.1007/s10802-010-9434-x 20623175

[55] Rønning JA, Handegaard BH, Sourander A, Mørch W. The Strengths and Difficulties Self-Report Questionnaire as a screening instrument in Norwegian community samples. European Child & Adolescent Psychiatry. 2004;13(2):73–82. Language: English. Entry Date: 20070101. Revision Date: 20091218. Publication Type: journal article. 15103532 10.1007/s00787-004-0356-4

[56] Leeuwen KV, Meerschaert T, Bosmans G, De Medts L, Braet C. The strengths and difficulties questionnaire in a community sample of young children in flanders. European Journal of Psychological Assessment. 2006;22(3):189–97. 10.1027/1015-5759.22.3.189

[57] Brown TA. Confirmatory factor analysis for applied research: Guilford Publications; 2015.

[58] Statistisk Sentralbyrå S. Barnevern https://www.ssb.no/statistikkbanken/selectvarval/saveselections.asp2014 [cited 2013 01.01.].

[59] Lehmann S, Breivik K, Heiervang E, Havik T, Havik O. Reactive Attachment Disorder and Disinhibited Social Engagement Disorder in School-Aged Foster Children—A Confirmatory Approach to Dimensional Measures. J Abnorm Child Psychol. 2015:1–13. 26126635 10.1007/s10802-015-0045-4 PMC4785216

