# Supplementary material for: The internal structure of foster-parent completed SDQ for school-aged children
Source: PLoS One. 2017 Jun 30;12(6):e0176625. doi: 10.1371/journal.pone.0176625 (PMC5493288; doi:10.1371/journal.pone.0176625)
Supplement: S1 Table — (DOCX) [file pone.0176625.s001.docx]

|  | 1 | 2 | 3 | 4 | 5 | 6 | 7 | 8 | 9 | 10 | 11 | 12 | 13 | 14 | 15 | 16 | 17 | 18 | 19 | 20 | 21 | 22 | 23 | 24 |
| --- | --- | --- | --- | --- | --- | --- | --- | --- | --- | --- | --- | --- | --- | --- | --- | --- | --- | --- | --- | --- | --- | --- | --- | --- |
| 1 Consid |  |  |  |  |  |  |  |  |  |  |  |  |  |  |  |  |  |  |  |  |  |  |  |  |
| 2 Restless | -.44 |  |  |  |  |  |  |  |  |  |  |  |  |  |  |  |  |  |  |  |  |  |  |  |
| 3 Somatic | -.21 | .26 |  |  |  |  |  |  |  |  |  |  |  |  |  |  |  |  |  |  |  |  |  |  |
| 4 Shares | .53 | -.20 | -.25 |  |  |  |  |  |  |  |  |  |  |  |  |  |  |  |  |  |  |  |  |  |
| 5 Tantrum | -.51 | .49 | .44 | -.41 |  |  |  |  |  |  |  |  |  |  |  |  |  |  |  |  |  |  |  |  |
| 6 Loner | -.23 | .26 | .10 | -.25 | .38 |  |  |  |  |  |  |  |  |  |  |  |  |  |  |  |  |  |  |  |
| **7 Obeys** | -.57 | .41 | .22 | -.30 | .62 | .30 |  |  |  |  |  |  |  |  |  |  |  |  |  |  |  |  |  |  |
| 8 Worries | -.29 | .40 | .364 | -.27 | .56 | .37 | .31 |  |  |  |  |  |  |  |  |  |  |  |  |  |  |  |  |  |
| 9 Caring | .73 | -.28 | -.15 | .60 | -.34 | -.07 | -.34 | -.29 |  |  |  |  |  |  |  |  |  |  |  |  |  |  |  |  |
| 10 Fidgety | -.52 | .85 | .32 | -.36 | .54 | .37 | .52 | .44 | -.34 |  |  |  |  |  |  |  |  |  |  |  |  |  |  |  |
| **11 Friend** | -.36 | .15 | .16 | -.24 | .37 | .49 | .37 | .34 | -.21 | .18 |  |  |  |  |  |  |  |  |  |  |  |  |  |  |
| 12 Fights | -.50 | .48 | .27 | -.40 | .62 | .47 | .48 | .26 | -.28 | .53 | .34 |  |  |  |  |  |  |  |  |  |  |  |  |  |
| 13 Unhappy | -.20 | .29 | .36 | -.30 | .64 | .43 | .27 | .67 | -.11 | .32 | .24 | .45 |  |  |  |  |  |  |  |  |  |  |  |  |
| **14 Popular** | -.39 | .33 | .21 | -.32 | .52 | .47 | .45 | .43 | -.26 | .38 | .43 | .51 | .32 |  |  |  |  |  |  |  |  |  |  |  |
| 15 Distract | -.39 | .55 | .24 | -.24 | .43 | .37 | .36 | .43 | -.28 | .56 | .28 | .52 | .36 | .38 |  |  |  |  |  |  |  |  |  |  |
| 16 Clingy | -.31 | .27 | .33 | -.32 | .33 | .14 | .20 | .34 | -.32 | .40 | .06 | .08 | .31 | .16 | .35 |  |  |  |  |  |  |  |  |  |
| 17 Kind | .60 | -.29 | -.07 | .59 | -.34 | -.13 | -.36 | -.17 | .62 | -.29 | -.13 | -.47 | -.20 | -.20 | -.19 | -.35 |  |  |  |  |  |  |  |  |
| 18 Lies | -.47 | .41 | .18 | -.39 | .48 | .27 | .47 | .38 | -.35 | .48 | .26 | .42 | .39 | .32 | .42 | .22 | -.32 |  |  |  |  |  |  |  |
| 19 Bullied | -.19 | .31 | .29 | -.20 | .49 | .49 | .44 | .46 | -.06 | .38 | .31 | .60 | .60 | .46 | .40 | .20 | -.24 | .35 |  |  |  |  |  |  |
| 20 Helpout | .42 | .03 | -.13 | .35 | .20 | -.06 | -.25 | -.17 | .53 | -.03 | -.03 | -.09 | -.03 | -.22 | -.10 | -.12 | .30 | -.14 | -.02 |  |  |  |  |  |
| **21 Reflects** | -.48 | .53 | .16 | -.29 | .40 | .31 | .55 | .31 | -.30 | .58 | .19 | .44 | .30 | .47 | .52 | .27 | -.20 | .50 | .46 | -.20 |  |  |  |  |
| 22 Steals | -.36 | .29 | .15 | -.49 | .46 | .28 | .41 | .34 | -.32 | .40 | .32 | .43 | .39 | .40 | .38 | .32 | -.26 | .71 | .39 | -.18 | .44 |  |  |  |
| 23 Oldbest | -.40 | .32 | .15 | -.44 | .38 | .65 | .24 | .30 | -.27 | .51 | .31 | .44 | .38 | .54 | .46 | .21 | -.21 | .38 | .36 | .02 | .41 | .38 |  |  |
| 24 Afraid | -.23 | .41 | .33 | -.22 | .43 | .27 | .24 | .53 | -.24 | .51 | .05 | .23 | .39 | .25 | .37 | .70 | -.28 | .21 | .33 | -.08 | .34 | .07 | .39 |  |
| **25 Attends** | -.42 | .47 | .23 | -.25 | .36 | .33 | .45 | .26 | -.31 | .53 | .20 | .37 | .29 | .40 | .80 | .25 | -.22 | .36 | .27 | -.13 | .52 | .41 | .35 | .24 |

S1 Table. Polychoric correlation matrix between items in the SDQ

Items in bold are reverse coded
